# 3′-Benzoyl-1′-methyl-4′-phenyl­spiro[acenaphthyl­ene-1(2*H*),2′-pyrrolidin]-2-one

**DOI:** 10.1107/S160053681004376X

**Published:** 2010-10-31

**Authors:** T. Augustine, Scholastica Mary Vithiya, S. Ignacimuthu, V. Ramkumar

**Affiliations:** a Department of Chemistry, D. G. Vaishnav College, Chennai 106, Tamil Nadu, India; b Department of Chemistry, Auxilium College, Vellore, Tamil Nadu, India; c Entomology Research Institute, Loyola College, Chennai 34, Tamil Nadu, India; d Department of Chemistry, Indian Institute of Technology Madras, Chennai 36, Tamil Nadu, India

## Abstract

In the title compound, C_29_H_23_NO_2_, the pyrrolidine ring adopts a twisted conformation about one of the C—N bonds. The acenaphthone ring (r.m.s. deviation = 0.025 Å) lies almost perpendicular to the pyrrolidine ring [dihedral angle = 88.08 (8)°]. The dihedral angle between the phenyl rings is 88.12 (11)°. In the crystal structure, weak C—H⋯π inter­actions connect the mol­ecules.

## Related literature

For background on 1,3-dipolar cyclo­additions, see: Grigg (1987 ▶); Huisgen (1988 ▶); Bridges *et al.* (1993 ▶); Padwa (1984 ▶). For a related structure, see: Augustine *et al.* (2007 ▶). For ring conformation analysis, see: Cremer & Pople (1975 ▶); Rao *et al.* (1981 ▶).
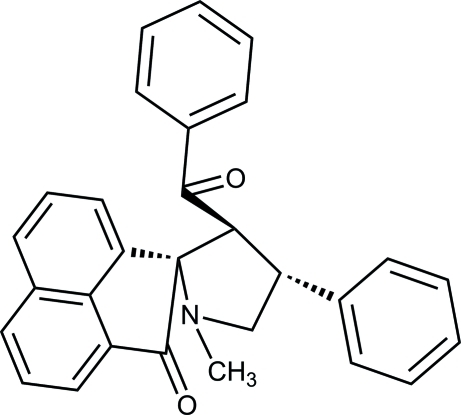


## Experimental

### 

#### Crystal data

C_29_H_23_NO_2_
*M* *_r_* = 417.48
Monoclinic, 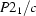

*a* = 8.6462 (4) Å
*b* = 15.8352 (8) Å
*c* = 16.7174 (8) Å
β = 99.827 (2)°
*V* = 2255.27 (19) Å^3^
*Z* = 4
Mo *K*α radiation
μ = 0.08 mm^−1^
*T* = 298 K
0.42 × 0.34 × 0.22 mm

#### Data collection

Bruker APEXII CCD area-detector diffractometer
Absorption correction: multi-scan (*SADABS*; Bruker, 2004 ▶) *T*_min_ = 0.969, *T*_max_ = 0.983
16782 measured reflections
5549 independent reflections
2944 reflections with *I* > 2σ(*I*)
*R*_int_ = 0.035

#### Refinement

*R*[*F* ^2^ > 2σ(*F* ^2^)] = 0.052
*wR*(*F* ^2^) = 0.142
*S* = 1.02
5549 reflections
290 parameters
H-atom parameters constrained
Δρ_max_ = 0.18 e Å^−3^
Δρ_min_ = −0.19 e Å^−3^

### 

Data collection: *APEX2* (Bruker, 2004 ▶); cell refinement: *APEX2* and *SAINT* (Bruker, 2004 ▶); data reduction: *SAINT* and *XPREP* (Bruker, 2004 ▶); program(s) used to solve structure: *SHELXS97* (Sheldrick, 2008 ▶); program(s) used to refine structure: *SHELXL97* (Sheldrick, 2008 ▶); molecular graphics: *ORTEP-3* (Farrugia, 1997 ▶); software used to prepare material for publication: *SHELXL97*.

## Supplementary Material

Crystal structure: contains datablocks global, I. DOI: 10.1107/S160053681004376X/hb5706sup1.cif

Structure factors: contains datablocks I. DOI: 10.1107/S160053681004376X/hb5706Isup2.hkl

Additional supplementary materials:  crystallographic information; 3D view; checkCIF report

## Figures and Tables

**Table 1:** Hydrogen-bond geometry (Å, °) *Cg*5 is the centroid of the C17–C22 ring.

*D*—H⋯*A*	*D*—H	H⋯*A*	*D*⋯*A*	*D*—H⋯*A*
C3—H3⋯*Cg*5^i^	0.93	2.85	3.638 (2)	144

Symmetry code: (i) 
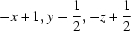
.

**Fig. 1.**
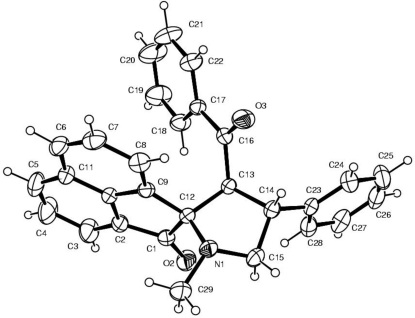
View of (I) with atoms represented as 30% probability ellipsoids.

**Fig. 2.**
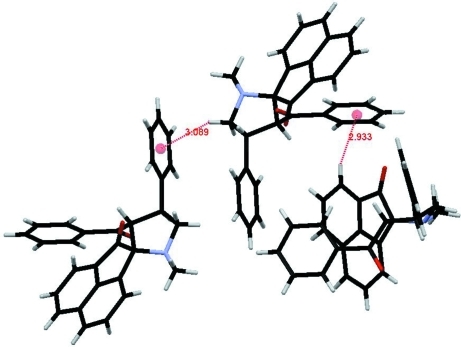
Packing diagram showing the C—H···π interactions.

## supplementary crystallographic information

### Comment

1,3-dipolar cycloadditions using azomethine ylides is conceptually the most simple and efficient method for the construction of saturated, nitrogen containing five-membered heterocycles (Padwa, 1984). Ylide generation, often performed *in situ* (Grigg, 1987), followed by cycloaddition with suitable dipolarophiles furnishes pyrrolidines and pyrroles in only one step from simple starting materials (Huisgen, 1988). 1,3-dipolar cycloadditions of azomethine ylides with olefinic and acetylenic dipolarophiles represent an important approach for the formation of pyrrolidines and pyrrolizines which are prevalent in a variety of biologically active compounds (Bridges *et al.*, 1993). In view of this we have determined the structure of the title compound.

In the title compound C_29_H_23_NO_2_, the C—O bond distance (1.21 Å) of the carbonyl group in the benzoyl moiety indicates n-p overlap. The bond angles and dihedral angle of C9—C12—C1 (101.69 Å) of the acenapthone ring indicate it to be in a plane nearly perpendicular to the pyrrolidine ring. The sum of the angles around N-atom of the pyrrolidine ring accounts for 338.78°. This indicates that the structure approaches pyramidal shape. The study of torsion angle, asymmetry parameters and least-square plane calculation shows that the pyrrolidine ring adopts a envelope conformation and puckered, Q_2_ = 0.4030 (18) Å, φ = 333.0 (3)° (Cremer & Pople, 1975). The Pseudorotation parameter P and τ are 136.4 (1)° and 43.71 (1)° respectively (Rao *et al.*, 1981) showing that C15 and N1 are twisted and puckered.

The crystal structure is stabilized by weak C—H···π interactions.

### Experimental

A mixture of chalcone [1,3-diphenyl-2-propen-1-one] (0.40 g, 2 mmol), acenaphthenequinone (0.36 g, 2 mmol), sarcosine (0.17 g, 2 mmol) and methanol (25 ml) was heated for four hours using oil bath using a dimmerstat at a temperature of 40° C. The reaction mixture was cooled to room temperature and poured into ice-cold water. The solid mass obtained was filtered, washed with water, dried and colourless blocks of (I) were obtained by recrystallization using acetone as solvent by slow evaporation method.

### Refinement

H atoms were positioned geometrically and refined using riding model,with C—H = 0.93 Å and *U*_iso_(H) = 1.2*U*_eq_(C) for aromatic C—H, C—H = 0.97 Å and *U*_iso_(H) = 1.2*U*_eq_(C) for CH_2_, C—H = 0.96 Å and *U*_iso_(H) = 1.5*U*_iso_(C) for CH_3_.

### Crystal data

**Table:**

C_29_H_23_NO_2_	*F*(000) = 880
*M**_r_* = 417.48	*D*_x_ = 1.230 Mg m^−^^3^
Monoclinic, *P*2_1_/*c*	Mo *K*α radiation, λ = 0.71073 Å
Hall symbol: -P 2ybc	Cell parameters from 3350 reflections
*a* = 8.6462 (4) Å	θ = 2.5–25.3°
*b* = 15.8352 (8) Å	µ = 0.08 mm^−^^1^
*c* = 16.7174 (8) Å	*T* = 298 K
β = 99.827 (2)°	Block, colourless
*V* = 2255.27 (19) Å^3^	0.42 × 0.34 × 0.22 mm
*Z* = 4	

### Data collection

**Table:**

Bruker APEXII CCD area-detector diffractometer	5549 independent reflections
Radiation source: fine-focus sealed tube	2944 reflections with *I* > 2σ(*I*)
graphite	*R*_int_ = 0.035
φ and ω scans	θ_max_ = 28.3°, θ_min_ = 1.8°
Absorption correction: multi-scan (*SADABS*; Bruker, 2004)	*h* = −9→11
*T*_min_ = 0.969, *T*_max_ = 0.983	*k* = −18→20
16782 measured reflections	*l* = −22→22

### Refinement

**Table:**

Refinement on *F*^2^	Primary atom site location: structure-invariant direct methods
Least-squares matrix: full	Secondary atom site location: difference Fourier map
*R*[*F*^2^ > 2σ(*F*^2^)] = 0.052	Hydrogen site location: inferred from neighbouring sites
*wR*(*F*^2^) = 0.142	H-atom parameters constrained
*S* = 1.01	*w* = 1/[σ^2^(*F*_o_^2^) + (0.0566*P*)^2^ + 0.296*P*] where *P* = (*F*_o_^2^ + 2*F*_c_^2^)/3
5549 reflections	(Δ/σ)_max_ < 0.001
290 parameters	Δρ_max_ = 0.18 e Å^−^^3^
0 restraints	Δρ_min_ = −0.19 e Å^−^^3^

### Special details

**Table:**

Geometry. All e.s.d.'s (except the e.s.d. in the dihedral angle between two l.s. planes) are estimated using the full covariance matrix. The cell e.s.d.'s are taken into account individually in the estimation of e.s.d.'s in distances, angles and torsion angles; correlations between e.s.d.'s in cell parameters are only used when they are defined by crystal symmetry. An approximate (isotropic) treatment of cell e.s.d.'s is used for estimating e.s.d.'s involving l.s. planes.
Refinement. Refinement of *F*^2^ against ALL reflections. The weighted *R*-factor *wR* and goodness of fit *S* are based on *F*^2^, conventional *R*-factors *R* are based on *F*, with *F* set to zero for negative *F*^2^. The threshold expression of *F*^2^ > σ(*F*^2^) is used only for calculating *R*-factors(gt) *etc*. and is not relevant to the choice of reflections for refinement. *R*-factors based on *F*^2^ are statistically about twice as large as those based on *F*, and *R*-factors based on ALL data will be even larger.

### Fractional atomic coordinates and isotropic or equivalent isotropic displacement parameters (Å^2^)

**Table:**

	*x*	*y*	*z*	*U*_iso_*/*U*_eq_	
C1	0.2530 (2)	0.32056 (11)	0.28710 (10)	0.0505 (5)	
C2	0.3044 (2)	0.27558 (11)	0.21910 (10)	0.0523 (5)	
C3	0.4124 (3)	0.21283 (14)	0.21511 (14)	0.0848 (7)	
H3	0.4681	0.1887	0.2621	0.102*	
C4	0.4365 (4)	0.18600 (16)	0.13784 (16)	0.1013 (9)	
H4	0.5112	0.1445	0.1344	0.122*	
C5	0.3546 (3)	0.21865 (15)	0.06826 (14)	0.0845 (7)	
H5	0.3733	0.1985	0.0185	0.101*	
C6	0.1491 (3)	0.32230 (14)	0.00346 (11)	0.0643 (6)	
H6	0.1588	0.3072	−0.0492	0.077*	
C7	0.0454 (2)	0.38280 (14)	0.01609 (11)	0.0631 (6)	
H7	−0.0157	0.4080	−0.0288	0.076*	
C8	0.0257 (2)	0.40942 (12)	0.09475 (11)	0.0549 (5)	
H8	−0.0478	0.4506	0.1012	0.066*	
C9	0.11632 (19)	0.37362 (10)	0.16061 (9)	0.0413 (4)	
C10	0.2211 (2)	0.31009 (10)	0.14725 (9)	0.0430 (4)	
C11	0.2423 (2)	0.28227 (12)	0.06998 (10)	0.0555 (5)	
C12	0.13015 (19)	0.38914 (10)	0.25123 (9)	0.0414 (4)	
C13	0.19337 (19)	0.47786 (10)	0.28373 (9)	0.0402 (4)	
H13	0.2961	0.4699	0.3181	0.048*	
C14	0.0763 (2)	0.50907 (11)	0.33801 (10)	0.0497 (5)	
H14	−0.0009	0.5458	0.3051	0.060*	
C15	−0.0067 (2)	0.42866 (13)	0.35599 (12)	0.0637 (6)	
H15A	0.0552	0.3972	0.4000	0.076*	
H15B	−0.1090	0.4407	0.3697	0.076*	
C16	0.2127 (2)	0.53926 (11)	0.21632 (10)	0.0447 (4)	
C17	0.35855 (19)	0.53647 (11)	0.17984 (9)	0.0437 (4)	
C18	0.4852 (2)	0.48560 (13)	0.20867 (12)	0.0612 (5)	
H18	0.4812	0.4498	0.2524	0.073*	
C19	0.6179 (3)	0.48749 (17)	0.17315 (16)	0.0906 (8)	
H19	0.7031	0.4531	0.1929	0.109*	
C20	0.6240 (3)	0.53984 (19)	0.10893 (17)	0.1026 (9)	
H20	0.7140	0.5412	0.0854	0.123*	
C21	0.5004 (3)	0.58984 (18)	0.07900 (14)	0.0889 (8)	
H21	0.5055	0.6248	0.0348	0.107*	
C22	0.3676 (2)	0.58877 (13)	0.11399 (11)	0.0625 (5)	
H22	0.2831	0.6233	0.0934	0.075*	
C23	0.1560 (2)	0.55919 (11)	0.41019 (11)	0.0495 (5)	
C24	0.1259 (3)	0.64403 (13)	0.41745 (13)	0.0690 (6)	
H24	0.0502	0.6700	0.3791	0.083*	
C25	0.2064 (3)	0.69126 (16)	0.48084 (18)	0.0879 (8)	
H25	0.1839	0.7484	0.4845	0.105*	
C26	0.3172 (3)	0.65544 (18)	0.53761 (16)	0.0827 (7)	
H26	0.3726	0.6878	0.5794	0.099*	
C27	0.3463 (3)	0.57130 (17)	0.53253 (13)	0.0767 (7)	
H27	0.4204	0.5456	0.5719	0.092*	
C28	0.2670 (2)	0.52382 (14)	0.46946 (12)	0.0649 (6)	
H28	0.2890	0.4665	0.4669	0.078*	
C29	−0.0869 (3)	0.29814 (14)	0.28011 (14)	0.0825 (7)	
H29A	−0.0220	0.2644	0.3202	0.124*	
H29B	−0.0912	0.2728	0.2276	0.124*	
H29C	−0.1908	0.3015	0.2928	0.124*	
N1	−0.02068 (18)	0.38299 (10)	0.27962 (9)	0.0565 (4)	
O2	0.29920 (19)	0.30958 (9)	0.35906 (7)	0.0752 (4)	
O3	0.10953 (17)	0.58979 (9)	0.19095 (8)	0.0704 (4)	

### Atomic displacement parameters (Å^2^)

**Table:**

	*U*^11^	*U*^22^	*U*^33^	*U*^12^	*U*^13^	*U*^23^
C1	0.0700 (12)	0.0418 (10)	0.0407 (10)	0.0002 (9)	0.0121 (8)	0.0050 (8)
C2	0.0703 (12)	0.0418 (10)	0.0463 (10)	0.0078 (10)	0.0138 (9)	0.0029 (8)
C3	0.1170 (19)	0.0658 (15)	0.0715 (14)	0.0422 (14)	0.0160 (13)	0.0069 (12)
C4	0.135 (2)	0.0850 (18)	0.0903 (19)	0.0531 (17)	0.0388 (17)	−0.0077 (15)
C5	0.1157 (19)	0.0786 (16)	0.0668 (15)	0.0179 (15)	0.0375 (14)	−0.0189 (13)
C6	0.0806 (15)	0.0741 (15)	0.0382 (10)	−0.0217 (12)	0.0106 (10)	−0.0067 (10)
C7	0.0625 (13)	0.0805 (15)	0.0414 (10)	−0.0169 (12)	−0.0050 (9)	0.0092 (10)
C8	0.0464 (10)	0.0618 (12)	0.0555 (11)	−0.0014 (9)	0.0062 (8)	0.0051 (9)
C9	0.0441 (9)	0.0410 (10)	0.0398 (9)	−0.0050 (8)	0.0098 (7)	0.0027 (7)
C10	0.0528 (10)	0.0395 (10)	0.0386 (9)	−0.0040 (8)	0.0129 (7)	−0.0022 (7)
C11	0.0707 (12)	0.0539 (12)	0.0455 (10)	−0.0071 (10)	0.0198 (9)	−0.0088 (9)
C12	0.0501 (10)	0.0378 (9)	0.0396 (8)	0.0014 (8)	0.0175 (7)	0.0017 (7)
C13	0.0441 (9)	0.0386 (9)	0.0415 (8)	0.0031 (7)	0.0172 (7)	0.0002 (7)
C14	0.0521 (10)	0.0487 (11)	0.0541 (10)	0.0063 (9)	0.0252 (8)	−0.0007 (8)
C15	0.0715 (13)	0.0652 (13)	0.0648 (12)	−0.0122 (11)	0.0412 (10)	−0.0122 (10)
C16	0.0509 (10)	0.0371 (10)	0.0484 (10)	0.0014 (9)	0.0153 (8)	−0.0006 (8)
C17	0.0460 (10)	0.0441 (10)	0.0435 (9)	−0.0115 (8)	0.0149 (7)	−0.0033 (8)
C18	0.0554 (12)	0.0624 (13)	0.0707 (12)	0.0027 (10)	0.0244 (10)	0.0129 (10)
C19	0.0621 (14)	0.1001 (19)	0.119 (2)	0.0144 (13)	0.0434 (14)	0.0281 (16)
C20	0.0779 (17)	0.128 (2)	0.118 (2)	0.0117 (18)	0.0625 (16)	0.0354 (19)
C21	0.0830 (17)	0.117 (2)	0.0747 (15)	−0.0113 (16)	0.0368 (13)	0.0311 (15)
C22	0.0587 (12)	0.0762 (14)	0.0537 (11)	−0.0109 (11)	0.0126 (9)	0.0105 (10)
C23	0.0562 (11)	0.0480 (11)	0.0510 (10)	0.0038 (9)	0.0284 (9)	−0.0048 (8)
C24	0.0813 (15)	0.0529 (13)	0.0771 (14)	0.0102 (11)	0.0261 (11)	−0.0052 (11)
C25	0.106 (2)	0.0577 (15)	0.109 (2)	−0.0035 (14)	0.0435 (17)	−0.0292 (15)
C26	0.0799 (17)	0.096 (2)	0.0821 (16)	−0.0169 (15)	0.0407 (14)	−0.0399 (15)
C27	0.0698 (14)	0.106 (2)	0.0576 (13)	0.0073 (13)	0.0194 (11)	−0.0129 (13)
C28	0.0769 (14)	0.0605 (13)	0.0610 (12)	0.0120 (11)	0.0228 (11)	−0.0084 (10)
C29	0.0991 (17)	0.0744 (16)	0.0854 (16)	−0.0376 (14)	0.0483 (13)	−0.0165 (12)
N1	0.0604 (10)	0.0557 (10)	0.0611 (10)	−0.0151 (8)	0.0322 (8)	−0.0083 (7)
O2	0.1194 (12)	0.0654 (9)	0.0392 (7)	0.0163 (8)	0.0087 (7)	0.0095 (6)
O3	0.0751 (9)	0.0600 (9)	0.0827 (10)	0.0249 (8)	0.0324 (7)	0.0237 (8)

### Geometric parameters (Å, °)

**Table:**

C1—O2	1.2136 (19)	C15—H15A	0.9700
C1—C2	1.473 (2)	C15—H15B	0.9700
C1—C12	1.565 (2)	C16—O3	1.219 (2)
C2—C3	1.373 (3)	C16—C17	1.492 (2)
C2—C10	1.402 (2)	C17—C18	1.379 (3)
C3—C4	1.409 (3)	C17—C22	1.390 (2)
C3—H3	0.9300	C18—C19	1.379 (3)
C4—C5	1.357 (3)	C18—H18	0.9300
C4—H4	0.9300	C19—C20	1.364 (3)
C5—C11	1.403 (3)	C19—H19	0.9300
C5—H5	0.9300	C20—C21	1.355 (3)
C6—C7	1.353 (3)	C20—H20	0.9300
C6—C11	1.408 (3)	C21—C22	1.375 (3)
C6—H6	0.9300	C21—H21	0.9300
C7—C8	1.418 (3)	C22—H22	0.9300
C7—H7	0.9300	C23—C28	1.375 (3)
C8—C9	1.361 (2)	C23—C24	1.378 (3)
C8—H8	0.9300	C24—C25	1.384 (3)
C9—C10	1.397 (2)	C24—H24	0.9300
C9—C12	1.519 (2)	C25—C26	1.353 (3)
C10—C11	1.406 (2)	C25—H25	0.9300
C12—N1	1.465 (2)	C26—C27	1.361 (3)
C12—C13	1.571 (2)	C26—H26	0.9300
C13—C16	1.519 (2)	C27—C28	1.379 (3)
C13—C14	1.551 (2)	C27—H27	0.9300
C13—H13	0.9800	C28—H28	0.9300
C14—C23	1.509 (2)	C29—N1	1.461 (2)
C14—C15	1.516 (3)	C29—H29A	0.9600
C14—H14	0.9800	C29—H29B	0.9600
C15—N1	1.454 (2)	C29—H29C	0.9600
			
O2—C1—C2	127.16 (17)	C14—C15—H15A	111.3
O2—C1—C12	124.49 (16)	N1—C15—H15B	111.3
C2—C1—C12	108.31 (14)	C14—C15—H15B	111.3
C3—C2—C10	119.66 (17)	H15A—C15—H15B	109.2
C3—C2—C1	133.22 (18)	O3—C16—C17	119.76 (15)
C10—C2—C1	107.11 (15)	O3—C16—C13	120.78 (15)
C2—C3—C4	118.1 (2)	C17—C16—C13	119.45 (15)
C2—C3—H3	120.9	C18—C17—C22	118.44 (17)
C4—C3—H3	120.9	C18—C17—C16	123.43 (15)
C5—C4—C3	122.3 (2)	C22—C17—C16	118.12 (16)
C5—C4—H4	118.9	C17—C18—C19	120.42 (19)
C3—C4—H4	118.9	C17—C18—H18	119.8
C4—C5—C11	121.20 (19)	C19—C18—H18	119.8
C4—C5—H5	119.4	C20—C19—C18	119.9 (2)
C11—C5—H5	119.4	C20—C19—H19	120.0
C7—C6—C11	120.06 (17)	C18—C19—H19	120.0
C7—C6—H6	120.0	C21—C20—C19	120.8 (2)
C11—C6—H6	120.0	C21—C20—H20	119.6
C6—C7—C8	122.84 (18)	C19—C20—H20	119.6
C6—C7—H7	118.6	C20—C21—C22	119.9 (2)
C8—C7—H7	118.6	C20—C21—H21	120.0
C9—C8—C7	118.87 (18)	C22—C21—H21	120.0
C9—C8—H8	120.6	C21—C22—C17	120.5 (2)
C7—C8—H8	120.6	C21—C22—H22	119.7
C8—C9—C10	118.02 (15)	C17—C22—H22	119.7
C8—C9—C12	132.51 (16)	C28—C23—C24	116.91 (19)
C10—C9—C12	109.46 (13)	C28—C23—C14	121.96 (17)
C9—C10—C2	113.33 (14)	C24—C23—C14	121.06 (18)
C9—C10—C11	124.21 (16)	C23—C24—C25	121.1 (2)
C2—C10—C11	122.45 (16)	C23—C24—H24	119.4
C5—C11—C10	116.29 (18)	C25—C24—H24	119.4
C5—C11—C6	127.74 (18)	C26—C25—C24	120.9 (2)
C10—C11—C6	115.96 (18)	C26—C25—H25	119.5
N1—C12—C9	112.92 (13)	C24—C25—H25	119.5
N1—C12—C1	114.40 (13)	C25—C26—C27	118.9 (2)
C9—C12—C1	101.69 (12)	C25—C26—H26	120.6
N1—C12—C13	102.93 (12)	C27—C26—H26	120.6
C9—C12—C13	116.96 (13)	C26—C27—C28	120.5 (2)
C1—C12—C13	108.34 (13)	C26—C27—H27	119.7
C16—C13—C14	113.29 (13)	C28—C27—H27	119.7
C16—C13—C12	113.06 (13)	C23—C28—C27	121.6 (2)
C14—C13—C12	105.31 (13)	C23—C28—H28	119.2
C16—C13—H13	108.3	C27—C28—H28	119.2
C14—C13—H13	108.3	N1—C29—H29A	109.5
C12—C13—H13	108.3	N1—C29—H29B	109.5
C23—C14—C15	116.74 (15)	H29A—C29—H29B	109.5
C23—C14—C13	112.37 (14)	N1—C29—H29C	109.5
C15—C14—C13	103.08 (14)	H29A—C29—H29C	109.5
C23—C14—H14	108.1	H29B—C29—H29C	109.5
C15—C14—H14	108.1	C15—N1—C29	115.24 (15)
C13—C14—H14	108.1	C15—N1—C12	107.82 (14)
N1—C15—C14	102.41 (14)	C29—N1—C12	115.72 (15)
N1—C15—H15A	111.3		
			
O2—C1—C2—C3	−0.6 (4)	C9—C12—C13—C14	131.26 (14)
C12—C1—C2—C3	177.3 (2)	C1—C12—C13—C14	−114.66 (14)
O2—C1—C2—C10	−179.13 (18)	C16—C13—C14—C23	−90.89 (18)
C12—C1—C2—C10	−1.20 (19)	C12—C13—C14—C23	145.06 (14)
C10—C2—C3—C4	0.7 (3)	C16—C13—C14—C15	142.59 (16)
C1—C2—C3—C4	−177.7 (2)	C12—C13—C14—C15	18.54 (17)
C2—C3—C4—C5	−1.5 (4)	C23—C14—C15—N1	−161.09 (15)
C3—C4—C5—C11	0.9 (4)	C13—C14—C15—N1	−37.41 (19)
C11—C6—C7—C8	0.6 (3)	C14—C13—C16—O3	−24.2 (2)
C6—C7—C8—C9	0.9 (3)	C12—C13—C16—O3	95.49 (18)
C7—C8—C9—C10	−2.1 (2)	C14—C13—C16—C17	156.88 (14)
C7—C8—C9—C12	177.34 (17)	C12—C13—C16—C17	−83.41 (18)
C8—C9—C10—C2	−177.88 (16)	O3—C16—C17—C18	175.23 (18)
C12—C9—C10—C2	2.6 (2)	C13—C16—C17—C18	−5.9 (2)
C8—C9—C10—C11	1.9 (3)	O3—C16—C17—C22	−3.9 (2)
C12—C9—C10—C11	−177.67 (16)	C13—C16—C17—C22	174.97 (16)
C3—C2—C10—C9	−179.55 (18)	C22—C17—C18—C19	0.6 (3)
C1—C2—C10—C9	−0.8 (2)	C16—C17—C18—C19	−178.6 (2)
C3—C2—C10—C11	0.7 (3)	C17—C18—C19—C20	−0.1 (4)
C1—C2—C10—C11	179.43 (16)	C18—C19—C20—C21	−0.5 (4)
C4—C5—C11—C10	0.5 (3)	C19—C20—C21—C22	0.7 (4)
C4—C5—C11—C6	179.7 (2)	C20—C21—C22—C17	−0.2 (4)
C9—C10—C11—C5	179.02 (18)	C18—C17—C22—C21	−0.4 (3)
C2—C10—C11—C5	−1.3 (3)	C16—C17—C22—C21	178.81 (19)
C9—C10—C11—C6	−0.4 (3)	C15—C14—C23—C28	55.7 (2)
C2—C10—C11—C6	179.36 (17)	C13—C14—C23—C28	−63.1 (2)
C7—C6—C11—C5	179.8 (2)	C15—C14—C23—C24	−127.57 (19)
C7—C6—C11—C10	−0.9 (3)	C13—C14—C23—C24	113.66 (18)
C8—C9—C12—N1	54.5 (2)	C28—C23—C24—C25	1.2 (3)
C10—C9—C12—N1	−126.08 (15)	C14—C23—C24—C25	−175.76 (18)
C8—C9—C12—C1	177.53 (18)	C23—C24—C25—C26	0.0 (3)
C10—C9—C12—C1	−3.02 (17)	C24—C25—C26—C27	−1.4 (4)
C8—C9—C12—C13	−64.7 (2)	C25—C26—C27—C28	1.6 (3)
C10—C9—C12—C13	114.74 (16)	C24—C23—C28—C27	−1.0 (3)
O2—C1—C12—N1	−57.4 (2)	C14—C23—C28—C27	175.89 (17)
C2—C1—C12—N1	124.57 (15)	C26—C27—C28—C23	−0.4 (3)
O2—C1—C12—C9	−179.48 (18)	C14—C15—N1—C29	175.49 (17)
C2—C1—C12—C9	2.53 (17)	C14—C15—N1—C12	44.53 (19)
O2—C1—C12—C13	56.7 (2)	C9—C12—N1—C15	−158.77 (15)
C2—C1—C12—C13	−121.27 (15)	C1—C12—N1—C15	85.56 (17)
N1—C12—C13—C16	−117.34 (15)	C13—C12—N1—C15	−31.75 (17)
C9—C12—C13—C16	7.1 (2)	C9—C12—N1—C29	70.5 (2)
C1—C12—C13—C16	121.15 (15)	C1—C12—N1—C29	−45.1 (2)
N1—C12—C13—C14	6.86 (16)	C13—C12—N1—C29	−162.45 (16)

### Hydrogen-bond geometry (Å, °)

**Table:**

*D*—H···*A*	*D*—H	H···*A*	*D*···*A*	*D*—H···*A*
C3—H3···Cg5^i^	0.93	2.85	3.638 (2)	144

Symmetry codes: (i) −*x*+1, *y*−1/2, −*z*+1/2.
